# Extracellular vesicles in the tumor microenvironment: old stories, but new tales

**DOI:** 10.1186/s12943-019-0980-8

**Published:** 2019-03-30

**Authors:** Liu Han, Eric W.-F. Lam, Yu Sun

**Affiliations:** 1 CAS Key Laboratory of Tissue Microenvironment and Tumor, Shanghai Institute of Nutrition and Health, Shanghai Institutes for Biological Sciences, University of Chinese Academy of Sciences, Chinese Academy of Sciences, Shanghai, 200031 China; 2 Department of Surgery and Cancer, Imperial College London, London, W12 0NN UK; 3 Department of Medicine and VAPSHCS, University of Washington, Seattle, WA 98195 USA

**Keywords:** Extracellular vesicles, Tumor microenvironment, Cancer biology, Therapeutic target, Clinical biomarker

## Abstract

Mammalian cells synthesize and release heterogeneous extracellular vesicles (EVs) which can be generally recognized as subclasses including exosomes, microvesicles (MVs), and apoptotic bodies (ABs), each differing in their biogenesis, composition and biological functions from others. EVs can originate from normal or cancer cells, transfer bioactive cargoes to both adjacent and distant sites, and orchestrate multiple key pathophysiological events such as carcinogenesis and malignant progression. Emerging as key messengers that mediate intercellular communications, EVs are being paid substantial attention in various disciplines including but not limited to cancer biology and immunology. Increasing lines of research advances have revealed the critical role of EVs in the establishment and maintenance of the tumor microenvironment (TME), including sustaining cell proliferation, evading growth suppression, resisting cell death, acquiring genomic instability and reprogramming stromal cell lineages, together contributing to the generation of a functionally remodeled TME. In this article, we present updates on major topics that document how EVs are implicated in proliferative expansion of cancer cells, promotion of drug resistance, reprogramming of metabolic activity, enhancement of metastatic potential, induction of angiogenesis, and escape from immune surveillance. Appropriate and insightful understanding of EVs and their contribution to cancer progression can lead to new avenues in the prevention, diagnosis and treatment of human malignancies in future medicine.

## Background

EVs are spherical bilayered small membranous vesicles generated by almost all cell types of mammalian organisms, although previous data indicated the presence of EVs also in lower eukaryotic and even prokaryotic lives [1, 2]. Studies in the late 1970s suggested that glycolipid-based EVs contribute to normal cell signaling, while the exact nature, function and biogenesis of EVs remained poorly understood in that era [3, 4]. As first reported in rat reticulocyte differentiation, multivesicular endosomes or multivesicular bodies (MVBs) release EVs into the surrounding microenvironment via fusion with plasma membrane of the parental cell [5, 6]. To date, three major subtypes of EVs can be classified according to an evolving consensus nomenclature: exosomes (30–120 nm in diameter), microvesicles (MVs, or ectosomes or microparticles, 0.1–1.0 μm) and apoptotic bodies (ABs, 0.8–5.0 μm) [7–9] (Table 1). Among them, ABs are less frequently engaged in intercellular communications, as after extracellular release they are usually engulfed by phagocytic cells [7].

**Table 1:** Major subtypes of EVs and their representative features

EV subtype	Subcellular origin	Regular diameter	Sedimentation force	Biogenetic mechanisms
Exosomes	Multivesicular bodies (MVBs)/ endosomes	30–120 nm	100,000 g	Rab proteins (i.e. Rab7, Rab11, Rab27A/B, Rab35), NSMase, ESCRTs, syndecan, syntenin, ATG12, tetraspanins
Microvesicles (MVs)/ectosomes /microparticles	Plasma membrane	100–1000 nm	10,000 g	ASMase, flippase, flippase and scramblase (TMEM16F), ARF6
Apoptotic bodies (ABs)	Apoptotic blebs	0.8–5.0 μm	2000 g	Annexin V, Caspase 3

Characterization of EV subtypes is based on recent scientific advances in EV biology [7, 124, 134, 135]

**Fig. 1:**
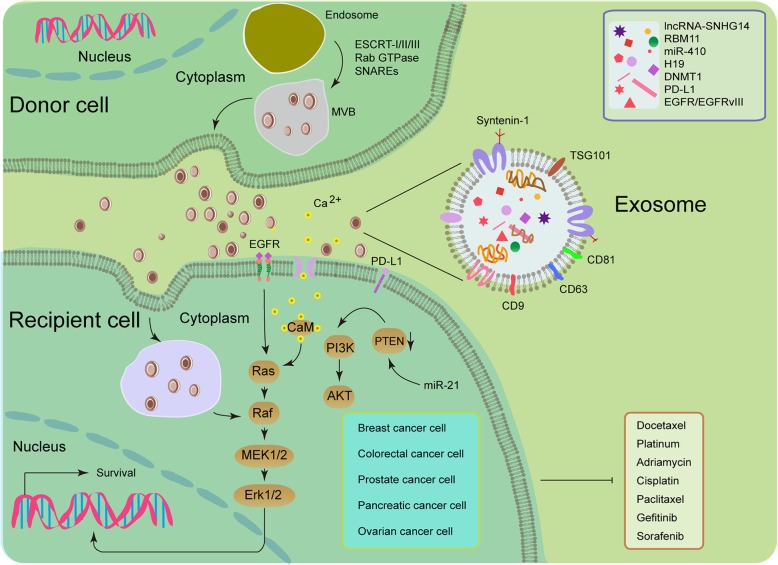
Illustrative diagram for exosome-mediated transfer of therapeutic resistance in the tumor microenvironment (TME). Drug-resistant (donor) cells may communicate with drug-sensitive (recipient) cells by the intercellular transfer of various types of EVs, such as exosomes (usually expressing tetraspanins such as CD9/63/81, TSG101 and syntenin-1), which are of endocytic origin [124]. Upon fusion of secretory multivesicular bodies (MVBs) with the plasma membrane, exosomes are released into the extracellular space. The initial steps of this process are usually modulated by the endosomal sorting complex required for transport (ESCRT) [125]. The mechanisms involved in the release of exosomes are also regulated by other protein families, such as Rab GTPases and SNARES [125, 126]. Once EVs reach the recipient cells, they may fuse with their plasma membrane or be internalized by the endocytic pathway. Exosomes may transfer miRNAs, lncRNAs, proteins (such as drug-efflux pumps), and other key players responsible for drug resistance, which allows de novo development or horizontal dissemination of cancer resistance traits to the recipient cell populations. For example, mesenchymal stem cell (MSC)-derived exosomes trigger the activation of calcium-dependent protein kinases and EGFR/Ras/Raf/Mek/Erk kinase cascade in gastric cancer cells, while polarized macrophages promote cisplatin resistance of gastric cancer cells by exosomal transfer of miR-21 which functionally activates PI3K/AKT signaling via down-regulation of PTEN in recipient cells [127, 128]

EVs carry various types of cargoes including membrane proteins, cytosolic proteins, lipids, diverse genetic materials such as DNA, mRNAs and non-coding RNAs such as microRNAs (miRNAs) [10–12]. EV components are biologically functional in recipient cells and highly variable depending on the cells of origin, while EVs containing various elements that can be generated under different conditions [12]. A causal role for EVs has been suggested in multiple physiological and pathological processes. In translational medicine, circulating EVs have also been of interest as a source for liquid biopsies, as EVs in body fluids carry a number of miRNA and proteins that hold the potential as novel cancer biomarkers [12]. Given the rapid research progression of EV biology, we hereby provide an updated profile of the state-of-the-art advancements in this blooming field, with a major focus on recent discoveries regarding the key activities of EVs, such as acquired resistance of cancer cells driven by exosomes in the tumor microenvironment (TME) (Fig. 1).

## Promoting cancer cell expansion

Accumulated genetic and epigenetic changes often activate the expression of oncogenes while silencing tumor suppressors during carcinogenesis. For example, malignant progression can be driven by an increasing number of secreted EVs that carry a truncated and oncogenic form of the epidermal growth factor receptor III (EGFRvIII), which enhance horizontal propagation of transformed phenotypes by transferring activated oncogenes between subsets of neoplastic cells [13]. A recent study analyzed the influence of oncogenic EGFRvIII on the profile of glioma EVs using isogenic cancer cell lines, and found that EGFRvIII reprograms the proteome and uptake of glioblastoma multiforme (GBM)-related EVs, suggesting substantial implications for biological activity of these EVs and properties relevant for their development as cancer biomarkers [14]. The transfer of the oncogenic phenotype via cancer cell-derived EVs also affects heterotypic cell types in the TME during pathological progression such as fibroblasts, endothelial cells and immune cells. For instance, EVs derived from cancer cells overexpressing a wild-type EGFR can result in angiogenesis by transferring the receptor to nearby endothelial cells and promoting their vascular endothelial growth factor (VEGF) expression, the latter can further induce activation of the key signaling receptor (VEGF receptor-2) in an autocrine manner [15]. In addition, the levels of cell-associated and circulating EV-carried tissue factor (TF, a primary cellular initiator of blood coagulation and a regulator of angiogenesis and metastasis) are correlated with the genetic status of cancer cells, such as an activated KRAS oncogene or a loss-of-function mutation of the p53 tumor suppressor, suggesting a causal link between cancer-associated coagulopathy, angiogenesis and malignant progression [16]. A new study disclosed that exposure of granulocytic HL-60 cells to EVs from oncogenic HRAS-driven cancer cells is responsible for a selective increase in TF pro-coagulant activity and interleukin 8 (IL-8) production, suggesting that these cells may represent a hitherto unrecognized reservoir of cancer-derived, EV-associated oncogenic genomic DNA in the circulation, and a potential novel platform for liquid biopsy in cancer clinics [17].

Exosomes from PC-1.0, a highly malignant pancreatic cell line, can be taken up by PC-1, a moderately malignant pancreatic line, and promote the proliferation rate of the latter [18]. Further studies identified a zinc transporter ZIP4 as the most upregulated exosomal protein in PC-1.0 cells and directly responsible for enhanced growth of recipient cells, with the potential to serve as a novel diagnostic marker for pancreatic cancer patients. In glioblastoma, a distinct EV uptake mechanism was recently uncovered, which involves a triple interaction between the chemokine receptor CCR8 on cancer cells, glycans exposed on EVs and the soluble ligand CCL18 as a bridging molecule connecting EVs to cancer cells [19]. Through such a mechanism, glioblastoma EVs promote cell proliferation and resistance to the alkylating agent temozolomide (TMZ).

In addition to proteins, EV-delivered miRNA molecules are also frequently involved in cancer cell expansion. For instance, miR-93-5p transferred by exosomes can promote the proliferation of recipient esophageal cancer cells and affect the expression of PTEN and its downstream proteins p21 and cyclin D1, enhancing the clinical risk of esophageal cancer [20]. A study analyzing exosomes derived from cancer-associated fibroblasts (CAFs) in oral squamous cell carcinoma (OSCC) unveiled that the miR-34a-5p/AXL axis can enhance OSCC progression via the AKT/GSK-3β/β-catenin signaling pathway, which induces the epithelial-mesenchymal-transition (EMT) to promote cancer cell growth and subsequent metastasis [21]. Thus, the miR-34a-5p/AXL axis confers aggressiveness on oral malignancies through an AKT-associated signaling cascade and represents a therapeutic target for OSCC.

## Resisting anticancer treatments

Cancer cells have evolved together with components of the surrounding microenvironment with strategies that counteract or circumvent cell apoptosis [22, 23]. Increasing evidence has proved that EVs are able to enhance the antiapoptotic capacity of neighboring cells. For example, MVs shed by MDA-MB-231 breast cancer (BCa) cells and U87 glioma cells can confer transformed characteristics of cancer cells including anchorage-independent growth and survival capability on normal fibroblasts and epithelial cells in nutrient-limiting conditions, a process mediated by cross-linking enzyme tissue transglutaminase (tTG) and dimerization of the substrate fibronectin (FN) at the EV surface [24]. Recent data suggested the involvement of EVs in acquired resistance of melanoma to BRAF inhibition and induced apoptosis by transporting a truncated but functional form of ALK, which activate the MAPK signaling pathway in target cells [25]. Human umbilical cord mesenchymal stem cell-derived EVs (MSC-EVs) can protect against ischemia-reperfusion injury (IRI)-induced hepatic apoptosis by reducing neutrophil infiltration and mitigating oxidative stress in hepatic tissue in vivo [26]. EVs from triple-negative breast cancer (TNBC) cells are capable of inducing proliferation and drug resistance of non-tumorigenic breast cells, a process mediated by expression change of genes and miRNAs correlated with development of malignant phenotypes [27]. In human epidermal growth factor receptor 2 (HER2) positive and trastuzumab-resistant BCa cells, expression of lncRNA-small nucleolar RNA host gene 14 (SNHG14) was higher than in parental cells, with lncRNA-SNHG14 packed into exosomes and transmitted to sensitive cells to disseminate trastuzumab resistance [28].

The transcript of DNA methyltransferase 1 (DNMT1) is highly enriched in exosomes from ovarian cancer cell-derived conditioned medium, and co-incubation with such exosomes rendered recipient cells resistant to cisplatin treatment, suggesting a critical role of exosomal DNMT1 in drug resistance of ovarian cancer [29]. Comprehensive analysis of established synovial sarcoma cell lines indicated that miR-761 putatively targets three proteins including thyroid hormone receptor interactor 6 (TRIP6), lamin A/C (LMNA), and NAD-dependent protein deacetylase sirtuin-3 (SIRT3), while knockdown of each protein can confer increased resistance to chemotherapeutic agents, implying miR-761 as a drug resistance biomarker and potential therapeutic target in future sarcoma clinics [30]. Importantly, a new study disclosed that apoptotic GBM cells can paradoxically enhance the proliferation and therapy resistance of surviving cancer cells by releasing apoptotic extracellular vesicles (apoEVs) that are enriched with various components of spliceosomes, while apoEVs alter RNA splicing in recipient cells and promote their drug resistance as well as migration capacity [31]. Specifically, RBM11 is a treatment-induced splicing factor upregulated in cancer cells and shed with EVs upon apoptosis induction, while once internalized in recipient cells RBM11 can switch MDM4 and cyclin D1 splicing toward the expression of more oncogenic variants. Further, the motility behavior of cancer cells expressing AXL, a receptor tyrosine kinase, can be elicited by Gas6-bearing ABs after treatment with apoptosis-inducing therapeutics that eliminate a portion of cancer cells, while such ABs substantially enhance the invasive and metastatic capability of surviving cell subsets [32].

Human umbilical cord MSC-derived EVs (hUCMSC-EV) are able to promote lung cancer cell growth and prevent their apoptosis, while the hUCMSC-EV-transmitted miR-410 mediates reduced PTEN expression [33]. The study revealed intercellular communications between MSCs and cancer cells via MSC-EV-miRNA and suggested that hUCMSC-EVs may be clinically employed as a new therapeutic option to minimize unwanted side effects. CAFs are an abundant and heterogeneous stromal cell subpopulation in the TME and are actively involved in cancer progression. A new study unclosed that colorectal cancer (CRC)-associated CAFs promote the stemness and chemoresistance of CRC by transferring exosomal H19, an imprinted maternally expressed transcript that can activate the β-catenin pathway as a competing endogenous RNA sponge for miR-141, which can otherwise inhibit the stemness of CRC cells [34]. The data indicate that CAFs of the CRC stroma contributes to malignant development and chemoresistance by producing H19 positive exosomes.

In contrast to the vast majority of anti-apoptosis data of EVs reported by the main body of literature, however, a recent investigation highlighted that exosomes derived from natural killer (NK) cells exert cytotoxic effects on B16F10 melanoma cells and thus warrant further development as a potential immunotherapeutic strategy for cancer medicine, suggesting the complexity of biological functions of TME-derived EVs [35].

## Remodeling metabolic activity

Cancer cells display a remarkable metabolic plasticity to generate the energy and meet the biosynthetic requirements to support their active proliferation and metastatic dissemination in a poorly oxygenated and nutrient-deprived TME [36, 37]. Many studies have demonstrated the presence of a metabolic symbiosis exists between cancer cells and the surrounding stroma. For instance, CAFs manifest increased anaerobic glycolytic activity in response to stimulation from cancer epithelial cells, causing the release of lactate and pyruvate, energy metabolites resulting from aerobic glycolysis and subsequently used by adjacent cancer cells in the mitochondrial TCA cycle to promote energy production and proliferative capacity, a phenomenon termed the “reverse Warburg effect” [38].

Proteins implicated in metabolism are among the most frequently identified proteins in EVs, although these vesicles also contain miRNA that are known to target proteins implicated in metabolic activities [39, 40]. Metabolism of fatty acid (FA) is emerging as a critical process for tumor progression, and FA metabolism can be modulated through intrinsic gene expression changes of cancer cells or intracellular communication within the local microenvironment, whereby EVs play an important role in remodeling FA metabolism [41]. Fatty acid synthase (FASN), a key enzyme biologically involved in the de novo synthesis of FAs, is one of the most frequently identified proteins in EVs [39]. Indeed, not only the protein but also the mRNA of FASN has been identified in prostate cancer (PCa) cell-derived EVs [42], implying a possible role of these EVs in lipogenesis of cancer cells. A recent study focusing on the CAF-derived exosomes (CDEs) disclosed inhibition of mitochondrial oxidative phosphorylation by CDEs, which contain intact metabolites such as amino acids, TCA-cycle intermediates and metabolites required for lipid synthesis such as acetate, materials that are avidly used by cancer cells for central carbon metabolism and promoting cellular proliferation [43]. These data indicate that EVs are able to supplement lipogenic substrates to recipient cells in the TME, a trait highly relevant to pathological exacerbation as malignant cells essentially need these building blocks for continuous proliferation.

Besides functional involvement in lipogenesis, EVs are emerging as a novel mechanism to allow FA transport via intracellular delivery and across cell membranes. Albumin is usually required to transport FA molecules through systemic circulation, but other intracellular carriers including fatty acid binding proteins (FABPs) are required in the course of internalization [44]. However, a number of studies have found that EVs also transport FAs [45]. Various forms of FAs are transported by EVs, although they are enriched in saturated FAs rather than monounsaturated and polyunsaturated FAs. Specifically, EV-carried FAs can be generated from phospholipids through phospholipase activities within the vesicles themselves, while they also originate directly from parental cells, as the quantity of FAs found within EVs is greater than the amount that could be generated from their own phospholipids [46].

It is noteworthy that FABPs, the key extracellular and intracellular FA transporters, are abundantly present in EVs released by multiple cell types (EVpedia database, [39]). Another membrane-associated FA transporter, CD36, was found in macrophage-derived EVs and is implicated in the control of EV uptake [47, 48]. Once internalized, FAs are converted into fatty acyl-CoAs which are transported by acyl-CoA binding proteins (ACBPs), molecules identified in hepatocellular carcinoma-associated EVs [49–51]. However, the specific functions of these vesicular transporters in cancer cells remains to be established.

EVs are implicated in not only lipid synthesis but also FA mobilization and use as an energy source by fatty acid oxidation (FAO), a process that requires delivery of FAs into the mitochondria and is catalyzed by carnitine palmitoyltransferase 1A (CPT1A), which transfers the acyl group of a fatty acyl-CoA from coenzyme A to carnitine [52]. Therefore, carnitine is a critical metabolite required for FAO. Interestingly, a recent study reported that EVs from PCa patients are enriched in carnitine, suggesting enhanced FA transport to mitochondria of PCa cells [53].

FAO can be modulated by peroxisome proliferator-activated receptors (PPARs), while both the protein and mRNA of PPAR isoforms have been identified in the cancer cell-derived EVs [54–56]. Together, these data suggest that the impact of EVs on FAO is likely multifactorial, and subject to regulation by the transport of the metabolites, substrates and enzymes essential for FAO.

Among various metabolic branches affected by EVs, the sugar-related pathways also merit substantial attention. A study comparing the exosome-associated proteomics of a non-aggressive EVs and aggressive hepatocellular carcinoma cell lines uncovered that aggressive cell-derived EVs are specifically enriched in glycolysis, gluconeogenesis and pentose phosphate pathways [57]. Thus, transfer of glycolytic enzymes via EVs may impact the metabolic profiling of recipient cells, a potential that is indeed possessed by glycolytic enzymes found within prostate acinar epithelial cell-derived EVs, which have a function for ATP generation when incubated with their substrates as a process required for EV uptake [58, 59]. As glycolytic enzymes are usually more abundant in the EVs released by aggressive cancer cells as part of the differentially expressed proteins (DEPs), these vesicles are likely to be more readily taken up by recipient cells, resulting in enhanced delivery of these metabolic drivers, a case well illustrated by hepatocellular carcinoma cells (HCC) [57]. Interestingly, the presence of such glycolytic enzymes in EVs is not necessarily correlated with a functional transfer, as a proteomic study performed on adipocyte EVs suggested that both glucose oxidation and lactic acid release remained largely unchanged in recipient cancer cells upon treatment with these vesicles [60].

On the other hand, glycolytic enzymes are among the most frequently identified proteins in the proteomics of EVs, which can display important energy-consuming functions by glycolytic conversion of saccharides such as glucose or fructose into ATP [61]. Tumor interstitial ATP levels exceed 1000 times of those in normal tissues of the same cell origin [62]. However, whether or not cancer cells exploit the abundant extracellular ATP remained unclear until a recent study revealed the capacity of cancer cells in internalizing ATP to perform multiple formerly unrecognized biological functions [62]. In some cases, glycolysis-promoted by cancer-derived exosomes could result in excess of extracellular ATP found in the interstitial space of TME. Such a glycolytic ATP production can not only limit the availability of glucose in a local TME niche but also elevate lactate levels since lactate dehydrogenase, an enzyme that catalyzes the conversion of the glycolytic end product pyruvate to lactate, is often identified in exosomes [61]. The high levels of lactates eventually restrain the proliferation and cytokine synthesis of human cytotoxic T cells, while promoting the expansion of myeloid-derived suppressor cells (MDSCs), the latter of critical implications in advanced tumor development [63–65]. Free ATP in the TME space is also responsible for enhanced amounts of extracellular adenosine generated by the sequential activities of ectonucleoside triphosphate diphosphohydrolase-1 (CD39) and 5′-nucleotidase (CD73). Both CD39 and CD73 are expressed by stromal cells in the TME and associated with cancer cell-derived exosomes, whereas adenosine is a potent immunoregulator and associated with cancer cell immune escape in an immunocompromised TME [66].

## Potentiating metastasis and establishing distant colonies

In the local TME, cancer cell motility is frequently subject to the influence of EVs. Autocrine secretion of EVs coated with FN-integrin/α5 complexes enhances persistent cell migration at the leading edge of human fibrosarcoma by reinforcing otherwise transient polarization states and enhancing cell adhesion assembly [67]. Interestingly, EVs derived from different tumor types have distinct integrin expression patterns which may determine organ-specific metastasis, and cancer cell-derived exosomes uptaken by organ-specific cells can prepare the pre-metastatic niche. For example, exosomal integrins α6β4 and α6β1 are associated with lung metastasis, while exosomal integrin αvβ5 is linked to liver metastasis, suggesting that exosomal integrins could be employed to predict organ-specific metastasis [68].

Bone marrow-derived cells (BMDCs) such as macrophages, neutrophils and mast cells contribute to malignant progression by modulating the pre-metastatic niche formation [69]. For example, EVs generated by highly metastatic melanoma cells can enhance the metastatic behavior of primary tumors by reprogramming bone marrow progenitors through the receptor tyrosine kinase Met, and induce vascular leakiness at pre-metastatic sites and reprogram bone marrow progenitors toward a pro-angiogenic phenotype dependent on c-Kit, the receptor tyrosine kinase Tie2 and Met [70]. Thus, EV production, intercellular transfer and education of bone marrow cells can potently accelerate tumor growth and metastasis, thus offering promise for new therapeutic directions in cancer treatment.

Pancreatic ductal adenocarcinomas (PDACs)-derived exosomes induce liver pre-metastatic niche establishment in naive mice, resulting in increased liver metastatic burden [71]. Specifically, uptake of PDAC-derived and migration inhibitory factor (MIF)-positive exosomes by Kupffer cells can increase the secretion of transforming growth factor β (TGF-β) and upregulation of fibronectin production by hepatic stellate cells, while such microenvironmental remodeling stimulates an influx of bone marrow-derived macrophages and provide a favorable niche for pancreatic metastasis in the liver. Upon contact with host stromal cell subpopulations particularly peritoneal mesothelial cells, fibroblasts, and endothelial cells, macrophages that have incorporated tumor-derived EVs (TEV-MΦs) can release membrane blebs containing these EVs, a process dependent on activation of caspase-3 in TEV-MΦs [72]. Scattered blebs taken by stromal cells promote transfer of cancer-derived RNA and proteins including TGF-β, activated Src, Wnt3, and HIF1α, components contributing to myofibroblastic changes in recipient stromal cells and eventually creating a pro-metastatic niche [72]. Thus, tumor-associated macrophages (TAMs) are able to transfer cancer-derived materials to surrounding stromal cells and induce a pro-metastatic microenvironment via generation of CAF-like cells.

Beyond BMDC-associated pre-metastatic niche formation, cancer cell-released EVs also directly contribute to the early steps of metastasis. Metastatic BCa cells, for instance, secrete EVs that carry miR-105, a potent modulator of migration through targeting the tight junction protein ZO-1 in endothelial cells [73]. Further, enhanced miR-105 expression in non-metastatic cancer cells induces metastasis and vascular permeability in distant organs, whereas inhibition of miR-105 in highly metastatic lesions alleviates such effects. In brain tumor, astrocyte-derived exosomes are responsible for intercellular transfer of PTEN-targeting microRNAs to metastatic cancer cells, while astrocyte-specific depletion of PTEN-targeting microRNAs or blockade of astrocyte exosome secretion rescues PTEN loss and suppresses brain metastasis in vivo [74]. Of note, two classes of cytotoxic agents widely employed in pre-operative (neoadjuvant) BCa therapy, namely taxanes and anthracyclines, can stimulate the release of tumor-derived EVs with remarkable pro-metastatic capacity [75]. These EVs are enriched in annexin A6 (ANXA6), a Ca^2+^-dependent factor that enhances NF-κB-dependent endothelial cell activation, CCL2 induction and Ly6C^+^CCR2^+^ monocyte expansion in the pulmonary niche to allow establishment of lung metastasis [75].

Recent analysis of the RNA components of EVs produced by PC3, a bone-metastatic PCa cell line, revealed that PCa EV-carried RNA molecules are substantially associated with cell surface signaling, cell-cell interaction, and protein translation [76]. Intercellular delivery of the RNA elements via PC3-derived EVs suggests communication mediated by RNA molecules in PCa EVs as a novel and important route to enhance bone metastasis, while targeting these EVs could offer a potentially feasible therapy for men at high risk of metastatic diseases. A recent study reported that pancreatic cancer-derived MVs are responsible for immune cell invasion regulated by CD36, a major mediator of the engulfment of MVs by myeloid immune cells, while MVs extravasation causes persistent infiltration of macrophages and cancer dissemination by metastasis in the TME [77]. Although special factors supporting liver metastasis of CRC remain poorly characterized, microRNA-21-5p was recently found to be highly enriched in CRC-derived exosomes and essential for creating a pro-inflammatory phenotype in liver and subsequent metastasis from primary CRC sites [78].

Among various cell types in the TME, adipocytes are attracting considerable attention due to the pathological link between obesity and cancer progression [41]. Adipocytes release a large number of bioactive molecules named adipokines including growth factors, hormones, cytokines and chemokines, whose balance is typically disturbed in obesity and associated complications [79, 80]. A former study reported that adipocytes cultivated with cancer cells display a modified phenotype with decreased lipid content (delipidation) and diminishing adipocyte markers, accompanied by overexpressed proteases and pro-inflammatory cytokines such as IL-6 and IL-1β, features that allow characterization of cells as cancer-associated adipocytes (CAA) [81]. Naïve adipocytes secrete exosomes enriched in proteins involved in lipid metabolism such as FAO-catalyzing enzymes, a signature specific to adipocytes that functionally enhances melanoma cell aggressiveness including migration and invasion through metabolic reprogramming in favor of FAO [60]. In obese animals and humans, both the number of adipocyte-secreted exosomes and their influence on FAO-dependent cell migration are increased, a fact that partially explain poorer prognosis of obese melanoma patients than their non-obese counterparts [60]. A new study highlighted that endothelial cells can transfer caveolin 1-containing EVs to adipocytes in vivo, which reciprocally release EVs containing proteins and lipids capable of modulating cellular signaling pathways to endothelial cells [82]. Hence, adipose tissue (AT)-derived EVs participate in the complex signaling network that exist among adipocytes, stromal vascular cells and, potentially, distal organs, which are frequently affected by cancer cells of metastatic potential.

## Inducing cancer-associated angiogenesis

In the course of tumors expansion, cells distant from blood vessels tend to become nutrient deficient, hypoxic or even necrotic [83]. Although angiogenesis is usually induced by soluble pro-angiogenic factors such as VEGF secreted by hypoxic and cancer cells to stimulate adjacent endothelial cells and recruit immune cells from bone marrow, recent studies discovered essential contributions of EVs to these processes. For instance, MVs produced by human cancer cells harboring activated EGFR can be absorbed by cultured endothelial cells which subsequently exhibit EGFR-dependent responses including activation of MAPK and Akt pathways, while the intercellular EGFR transfer is accompanied by the onset of VEGF expression in these endothelial cells and autocrine activation of VEGF receptor-2 [15]. Further, proteins and/or mRNAs carried by exosomes derived from the plasma of patients developing highly malignant GBM display a molecular signature correlated with the hypoxic status and aggressiveness of cancer cells [84]. Thus, the proteome and mRNA profiles of exosome closely reflect the oxygenation status of donor glioma cells, while the exosome-mediated transmission constitutes a potentially targetable driver of hypoxia-dependent intercellular signaling during GBM development.

A recent study found that miR-130a is delivered by exosomes from gastric cancer cells into human umbilical vein endothelial cells (HUVECs) to promote angiogenesis and tumor expansion through targeting c-MYB both in vivo and in vitro [85]. Hence, miR-130a packaged in exosomes of cancer cells serves as an angiogenesis driver, while targeting the expression or blocking the transmission of such exosomes might be a novel anti-angiogenic strategy for gastric malignancies. In contrast, exosomes from pancreatic cancer cells activate various gene expression in HUVECs, promote phosphorylation of Akt and ERK1/2 signaling molecules and tube formation via dynamin-dependent endocytosis, suggesting that pancreatic cancer cell-released exosomes may act as a novel angiogenesis stimulator [86]. In head and neck squamous cell carcinoma (HNSCC), exosomes are potent inducers of angiogenesis via phenotypic modification and functional reprogramming of endothelial cells [87]. Specifically, HNSCC-derived exosomes stimulate proliferation, migration and tube formation of HUVECs in vitro and promote vascular structure formation in vivo*,* playing an active role in tumor angiogenesis and may contribute to HNSCC metastasis. Of note, hepatocellular carcinoma cell HepG2-derived exosomes can be internalized by adipocytes, which consequently exhibit significantly changed transcriptomics, development of an inflammatory phenotype and enhanced capacity to induce angiogenesis and recruit macrophages in xenograft mice [88]. Intriguingly, the effects of the HepG2-exosomes on the lumen formation of HUVECs can be measured by imaging angiogenic activities, the degree of which is dependent on the number of exosomes related by HepG2 cells [89]. The soluble form of E-cadherin (sE-cad) is highly expressed in malignant ascites of ovarian cancer patients and can act as a potent inducer of angiogenesis via delivery by exosomes to heterodimerize with vein endothelial (VE)-cadherin on endothelial cells, a process that causes sequential activation of β-catenin and NF-κB signaling [90].

## Modulating immune responses in the TME

Cancer progression is intimately linked with chronic inflammation and involves dysregulated activity of immune cell subsets. Clinical and preclinical studies indicate that tumor-associated macrophages (TAMs) provide important pro-tumorigenic and survival factors, pro-angiogenic factors and extracellular matrix (ECM)-modifying enzymes [91]. Cancer cell-derived EVs promote the induction and persistence of inflammation that functionally contributes to disease progression [92].

Under hypoxic conditions, epithelial ovarian cancer (EOC) cell-derived exosomes deliver miRNAs to modify the polarization of M2 macrophages, eventually promoting EOC cell proliferation and migration, suggesting exosomes and associated miRNAs as potential targets for novel treatments of EOC or diagnostic biomarkers in ovarian cancer clinics [93, 94]. EVs harboring damage-associated molecular pattern (DAMP) molecules and acting as danger signals are released from injured or stressed tissues and contribute to the induction and persistence of inflammation [95], although the biological role of signaling via EV-associated DAMPs remains to be determined. In addition to EV-associated DAMPs, miRNAs can also interact with the single-stranded RNA-binding Toll-like receptor (TLR) family, a type of pattern recognition receptor [96]. As TLR signaling frequently activates the NF-kB complex and induces the secretion of pro-inflammatory cytokines, miRNAs, and other components transmitted through EVs, it may significantly enhance inflammation and promote cancer development. Specifically, BCa cell-derived exosomes can stimulate NF-кB activation in macrophages, resulting in secretion of diverse cytokines including IL-6, TNF-α, G-CSF and CCL2, while genetic depletion of Toll-like receptor 2 (TLR2) or MyD88, a critical signaling adaptor of the NF-кB pathway, completely abrogates the effect of tumor-derived exosomes [97]. Thus, BCa cells employ a distinct mechanism to induce pro-inflammatory activity of distant macrophages via circulating exosome generated during cancer progression.

Transfer of chronic lymphocytic leukemia (CLL)-derived exosomes or transmission of hY4, a non-coding Y RNA enriched in exosomes of CLL patient plasma, to monocytes can generate key CLL-associated phenotypes, including the release of cytokines CCL2, CCL4 and IL-6, and the expression of programmed cell death ligand 1 (PD-L1) [98]. Thus, exosome-mediated transfer of non-coding RNAs to monocytes contributes to cancer-associated inflammation and potential immune escape via PD-L1 upregulation.

In the settings of carcinogenesis, the immune system which initially restrict disease progression, is progressively disabled, as exacerbated by regulatory T cell (T_reg_)-mediated immune suppression and PD-L1-induced immune checkpoint activation in the TME [99, 100]. However, an emerging alternative mechanism of immunosurveillance deficiency involves the active release of immunosuppressive EVs from cancer cells. For instance, tumor-derived MVs can inhibit signaling and proliferation activated CD8(+) T cells, while inducing the expansion of CD4(+)CD25(+)FOXP3(+) Treg cells and enhancing their suppressor activity [101]. The data suggest that tumor-derived MVs induce immune suppression by promoting Treg cell expansion and the demise of antitumor CD8(+) effector T cells to allow tumor escape.

A new study disclosed that metastatic melanomas release EVs, mostly in the form of exosomes, which carry PD-L1 on their surface and suppress CD8 T cell function [102]. The study unmasked a novel mechanism by which cancer cells systemically dampen the immune system, and provided a rationale for application of exosomal PD-L1 as a predictor for anti-PD-1 therapy.

Beyond various T cell types, other immune cell lineages are also subject to the impact of EVs generated by cells in the TME. The proliferation, activation and cytotoxicity of NK cells can be affected by fetal liver MSC-derived exosomes, which deliver a regulatory molecule for TGF-β and result in the downstream TGF-β/Smad2/3 signaling in NK cells [103]. Hence, MSC-derived exosomes are able to regulate NK cell function through exosome-associated TGF-β, with the potential to compromise immunosurveillance.

## Concluding remarks and future directions

A timely and comprehensive landscape illustrating conceptual and technical milestones in cancer biology is summarized by Hanahan and Weinberg, allowing to clearly understand the hallmarks of cancer [104]. EVs represent a diverse category of cellular export products present in multiple types of biofluids and cell culture media. Although our knowledge of EVs continues to augment, it is far from complete. Experimental data accumulated since decades ago evidently suggests that EVs are critical for some, if not all, cancer hallmarks. To date, the field of EV research has attracted mounting interest from scientists and clinicians, and the number of investigations dissecting on the critical role of EVs in cancer biology keeps rising.

**Fig. 2:**
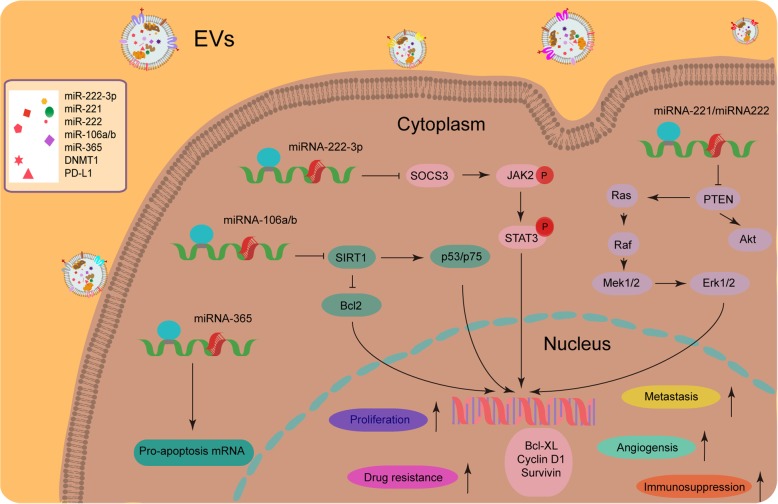
Multiple roles of EV-delivered cargoes such as microRNAs (miRNAs) in altering the phenotypes of recipient cancer cells and shaping a pathologically active tumor microenvironment (TME). Cancer cells and stromal cells utilize EVs such as exosomes to influence surrounding cells within the microenvironmental niche by transferring bioactive molecules including miRNAs. Sorting miRNAs to EVs is regulated by cell activation-dependent changes in miRNA levels within donor cells. Specifically, miRNA-365, miRNA-106a/b, miRNA-222-3p and miRNA-221/222 are not only overexpressed in donor cells but also enriched in their exosomes, and upon exosome-mediated transmission these miRNAs can significantly enhance resistance of recipient cancer cells against anticancer agents [129–133]. In addition, other malignant properties including but not limited to proliferation capacity, angiogenesis capability, metastatic potential and immunosurveillance evasion are also subject to the impact of EVs released by stromal or cancer cells in the TME

In cancer clinics, the great strength of liquid biopsy is the ability to provide pathological information before and during treatment for therapeutic design and evaluation. Over the past decade, circulating EVs are proved to be a reliable source of cancer-related molecules (typically miRNAs) with unique potential as biomarkers for many cancer types, including malignancies developed in liver, lung, pancreas, skin, breast, ovary, prostate and gastrointestinal tract [105]. EVs carry a large array of bioactive macromolecules that are indeed a sampling of the cytoplasmic or endosomal compartments, and functionally engaged in cell-to-cell paracrine signaling to change recipient cell phenotypes (Fig. 2). Due to their relative stability, increased concentration, and unique molecular signatures in cancer patients, EVs are emerging as a subject of intensive exploration for diagnostic and prognostic purposes in cancer medicine [106]. In addition to miRNAs, other EV cargo molecules, such as oncogenic mRNAs (including transcripts of fusion gene) and their splice variants, double-stranded DNA fragments (including cancer-driving gene mutants), various forms of lipids and lncRNAs, are gaining much attention as candidates for potential biomarkers of future clinical utility [105].

There are currently going advancements in EV subtype characterization, biofluid EV capturing and proteomic assessment technologies, as well as possible EV-based multiomics for cancer patient diagnostics [107, 108]. However, a universally accepted consensus regarding the standard nomenclature, technical isolation, purification strategy and biological composition of EV subtypes has yet to be established [109]. Even the current ‘state-of-the-art’ preparation methods are less than optimal [110].

Intriguingly, some studies found that EVs can also inhibit tumor progression, either by direct influence of the EV-carried protein and nucleic acid components or via antigen presentation to immune cells, the latter mediated by certain antigens expressed by the donor cells but simultaneously manifested by these cancer cell-derived EVs [111]. For example, dendritic cells (DCs) primed with rat glioblastoma cell-derived exosomes can induce a strong anticancer response and markedly increase median survival in glioblastoma-bearing rats when used in combination with α-galactosylceramide [112].

As natural carriers for diverse bioactive cargoes, EVs indeed have attracted increasing attention as potential vehicles for the delivery of many forms of therapeutic substances including mRNAs, miRNAs, lncRNAs, proteins, peptides and synthetic drugs [105]. Using either passive or active approaches, such therapeutically effective components can be loaded into EVs. The most common in vitro methods involve either passive loading through physically admixing pharmaceutical agents, as exemplified by acridine orange, curcumin, doxorubicin, or paclitaxel with isolated EVs, or active priming by techniques such as electroporation employed to deliver materials like oncogenic KRAS^G12D^-specific small interfering RNAs to cancer cells [113–116]. Alternatively, genetic engineering of EV-producing cells to overexpress proteins such as TNF-related apoptosis-inducing ligand (TRAIL), miRNAs such as miR-122 from an expression plasmid, or mRNA/protein molecules aimed to promote their enrichment in EVs, has been illustrated by some pilot studies [117–119]. Notably, EVs possess multiple advantages as drug delivery tools due to their excellent biocompatibility, low immunogenicity and innate capacity to interact with target cells, although restraints and challenges remain and warrant continued study to expand EV-related therapeutics to cancer clinics. For instance, identification of optimal EV donor cell type, preservation of EV structural integrity during agent loading and large-scale production, long term storage and maintenance of EV efficacy, all issues yet to be solved by emerging pipelines in scientific and industrial efforts [105].

Given the increasing lines of EV-associated studies, the field of EV biology requires more transparent reporting and documenting activities to support interpretation and replication of experiments. EV-TRACK, a crowdsourcing knowledgebase (http://evtrack.org) is recently established to allow centralization of EV biology and associated methodology to inspire authors, reviewers, editors and funders to put experimental guidelines into practice and increase research reproducibility [120, 121]. Vesiclepedia (http://www.microvesicles.org) is established as a web-based compendium of proteins, RNA, lipids and metabolites identified in EVs from both published and unpublished studies, with the data currently from 1254 EV investigations, 349,988 protein entries, 38,146 RNA entries and 639 lipid/metabolite entries [122]. There are also alternative or supplementary initiatives to characterize EVs, such as EVpedia and ExoCarta, two representative webdomains that facilitate researchers to handily upload proteomic lists of identified proteins of the EVs they are investigating [39, 123]. It is believed that widespread implementation by the EV scientific community is key to its success in the long run.

Despite the mounting advances, some EV-oriented questions remain unanswered and are subject to extensive studies in future. Cancer-associated EVs exert their systemic effects partly through transfer of various types of cargoes, resulting in the reprogramming of stromal cells, immune cells, and BMDCs in the surrounding TME. Are these activities mediated by a genetic or epigenetic mechanism? Are the consequences permanent or transient? Are the phenotypic alterations reversible or irreversible? It is possible to examine the role of EVs in vivo of genetic models in which EV dynamics can be monitored real time? How is the rate of EV secretion modulated by parental cells? Are EVs functionally complementary or redundant to soluble factors from the same cells? By solving these remaining, fascinating but essential issues with incremental inputs, we can imagine that EV biology will significantly help unravel the highly intricate nature of cancer and contribute to the development of improved diagnostics and therapies in prospective clinical oncology.

## Abbreviations

AB: Apoptotic body
ACBP: Acyl-CoA binding protein
ANXA6: Annexin A6
apoEV: Apoptotic extracellular vesicle
AT: Adipose tissue
BCa: Breast cancer
BMDC: Bone marrow-derived cell
CAA: Cancer-associated adipocyte
CAF: Cancer-associated fibroblast
CDE: CAF-derived exosome
CLL: Chronic lymphocytic leukemia
CPT1A: Carnitine palmitoyltransferase 1A
CRC: Colorectal cancer
DAMP: Damage-associated molecular pattern
DC: Dendritic cell
DEP: Differentially expressed protein
DNMT1: DNA methyltransferase 1
ECM: Extracellular matrix
EGFRvIII: Epidermal growth factor receptor III
EMT: Epithelial-mesenchymal-transition
EOC: Epithelial ovarian cancer
ESCRT: Endosomal sorting complex required for transport
EV: Extracellular vesicle
FA: Fatty acid
FABP: Fatty acid binding protein
FAO: Fatty acid oxidation
FASN: Fatty acid synthase
FN: Fibronectin
GBM: Glioblastoma multiforme
gDNA: Genomic DNA
HCC: Hepatocellular carcinoma cell
HER2: Human epidermal growth factor receptor 2
HNSCC: Head and neck squamous cell carcinoma
hUCMSC-EV: Human umbilical cord MSC-derived EV
HUVEC: Human umbilical vein endothelial cell
IL: Interleukin
IRI: Ischemia-reperfusion injury
LMNA: Lamin A/C
lncRNA: Long noncoding RNA
MDSC: Myeloid-derived suppressor cell
MIF: Migration inhibitory factor
miRNA: microRNAs
MSC: Mesenchymal stem cell
MSC-EV: Mesenchymal stem cell-derived EV
MV: Microvesicle
MVB: Multivesicular body
NK: Natural killer
OSCC: Oral squamous cell carcinoma
PCa: Prostate cancer
PDAC: Pancreatic ductal adenocarcinoma
PD-L1: Programmed cell death ligand 1
PPAR: Peroxisome proliferator-activated receptor
sE-cad: Soluble form of E-cadherin
SIRT3: Sirtuin-3
SNHG14: Small nucleolar RNA host gene 14
TAM: Tumor-associated macrophagesl
TEV-MΦ: Macrophages with incorporated tumor-derived EV
TF: Tissue factor
TGF-β: Transforming growth factor beta
TLR: Toll-like receptor
TME: Tumor microenvironment
TMZ: Temozolomide
TNBC: Triple-negative breast cancer
TRAIL: TNF-related apoptosis-inducing ligand
T_reg_: Regulatory T cell
TRIP6: Thyroid hormone receptor interactor 6
tTG: Tissue transglutaminase
VE: Vein endothelial
VEGF: Vascular endothelial growth factor

## References

[1] Ellis TN, Kuehn MJ (2010). Virulence and immunomodulatory roles of bacterial outer membrane vesicles. Microbiol Mol Biol Rev.

[2] Beveridge TJ (1999). Structures of gram-negative cell walls and their derived membrane vesicles. J Bacteriol.

[3] Esselman WJ, Miller HC (1977). Modulation of B cell responses by glycolipid released from antigen-stimulated T cells. J Immunol.

[4] Freimuth WW, Miller HC, Esselman WJ (1979). Soluble factors containing Thy-1 antigen shed from lymphoblastoid cells modulate in vitro plaque-forming cell response. J Immunol.

[5] Harding C, Heuser J, Stahl P (1983). Receptor-mediated endocytosis of transferrin and recycling of the transferrin receptor in rat reticulocytes. J Cell Biol.

[6] Pan BT, Johnstone RM (1983). Fate of the transferrin receptor during maturation of sheep reticulocytes in vitro: selective externalization of the receptor. Cell..

[7] Budnik V, Ruiz-Canada C, Wendler F (2016). Extracellular vesicles round off communication in the nervous system. Nat Rev Neurosci.

[8] Ludwig AK, De Miroschedji K, Doeppner TR, Borger V, Ruesing J, Rebmann V, Durst S, Jansen S, Bremer M, Behrmann E (2018). Precipitation with polyethylene glycol followed by washing and pelleting by ultracentrifugation enriches extracellular vesicles from tissue culture supernatants in small and large scales. J Extracell Vesicles.

[9] Tkach M, Thery C (2016). Communication by extracellular vesicles: where we are and where we need to Go. Cell..

[10] Milane L, Singh A, Mattheolabakis G, Suresh M, Amiji MM (2015). Exosome mediated communication within the tumor microenvironment. J Control Release.

[11] Sun Z, Yang S, Zhou Q, Wang G, Song J, Li Z, Zhang Z, Xu J, Xia K, Chang Y (2018). Emerging role of exosome-derived long non-coding RNAs in tumor microenvironment. Mol Cancer.

[12] Fujita Y, Yoshioka Y, Ochiya T (2016). Extracellular vesicle transfer of cancer pathogenic components. Cancer Sci.

[13] Al-Nedawi K, Meehan B, Micallef J, Lhotak V, May L, Guha A, Rak J (2008). Intercellular transfer of the oncogenic receptor EGFRvIII by microvesicles derived from tumour cells. Nat Cell Biol.

[14] Choi D, Montermini L, Kim DK, Meehan B, Roth FP, Rak J (2018). The impact of oncogenic EGFRvIII on the proteome of extracellular vesicles released from glioblastoma cells. Mol Cell Proteomics.

[15] Al-Nedawi K, Meehan B, Kerbel RS, Allison AC, Rak J (2009). Endothelial expression of autocrine VEGF upon the uptake of tumor-derived microvesicles containing oncogenic EGFR. Proc Natl Acad Sci U S A.

[16] Yu JL, May L, Lhotak V, Shahrzad S, Shirasawa S, Weitz JI, Coomber BL, Mackman N, Rak JW (2005). Oncogenic events regulate tissue factor expression in colorectal cancer cells: implications for tumor progression and angiogenesis. Blood..

[17] Chennakrishnaiah S, Meehan B, D'Asti E, Montermini L, Lee TH, Karatzas N, Buchanan M, Tawil N, Choi D, Divangahi M (2018). Leukocytes as a reservoir of circulating oncogenic DNA and regulatory targets of tumor-derived extracellular vesicles. J Thromb Haemost.

[18] Jin HY, Liu P, Wu YH, Meng XL, Wu MW, Han JH, Tan XD (2018). Exosomal zinc transporter ZIP4 promotes cancer growth and is a novel diagnostic biomarker for pancreatic cancer. Cancer Sci.

[19] Berenguer J, Lagerweij T, Zhao XW, Dusoswa S, van der Stoop P, Westerman B, de Gooijer MC, Zoetemelk M, Zomer A, Crommentuijn MHW (2018). Glycosylated extracellular vesicles released by glioblastoma cells are decorated by CCL18 allowing for cellular uptake via chemokine receptor CCR8. J Extracell Vesicles.

[20] Liu MX, Liao J, Xie M, Gao ZK, Wang XH, Zhang Y, Shang MH, Yin LH, Pu YP, Liu R (2018). miR-93-5p Transferred by Exosomes Promotes the Proliferation of Esophageal Cancer Cells via Intercellular Communication by Targeting PTEN. Biomed Environ Sci.

[21] Li YY, Tao YW, Gao S, Li P, Zheng JM, Zhang SE, Liang JF, Zhang YJ (2018). Cancer-associated fibroblasts contribute to oral cancer cells proliferation and metastasis via exosome-mediated paracrine miR-34a-5p. Ebiomedicine..

[22] Chen F, Zhuang X, Lin L, Yu P, Wang Y, Shi Y, Hu G, Sun Y (2015). New horizons in tumor microenvironment biology: challenges and opportunities. BMC Med.

[23] Sun Y (2015). Translational horizons in the tumor microenvironment: harnessing breakthroughs and targeting cures. Med Res Rev.

[24] Antonyak MA, Li B, Boroughs LK, Johnson JL, Druso JE, Bryant KL, Holowka DA, Cerione RA (2011). Cancer cell-derived microvesicles induce transformation by transferring tissue transglutaminase and fibronectin to recipient cells. Proc Natl Acad Sci U S A.

[25] Cesi G, Philippidou D, Kozar I, Kim YJ, Bernardin F, Van Niel G, Wienecke-Baldacchino A, Felten P, Letellier E, Dengler S (2018). A new ALK isoform transported by extracellular vesicles confers drug resistance to melanoma cells. Mol Cancer.

[26] Yao J, Zheng J, Cai J, Zeng K, Zhou C, Zhang J, Li S, Li H, Chen L, He L, et al. Extracellular vesicles derived from human umbilical cord mesenchymal stem cells alleviate rat hepatic ischemia-reperfusion injury by suppressing oxidative stress and neutrophil inflammatory response. FASEB J. 2018;33:1695–710. 10.1096/fj.201800131RR 30226809

[27] Ozawa PMM, Alkhilaiwi F, Cavalli IJ, Malheiros D, de Souza Fonseca Ribeiro EM, Cavalli LR. Extracellular vesicles from triple-negative breast cancer cells promote proliferation and drug resistance in non-tumorigenic breast cells. Breast Cancer Res Treat. 2018;172:713–23. 10.1007/s10549-018-4925-5 PMC6245099 30173296

[28] Dong H, Wang W, Chen R, Zhang Y, Zou K, Ye M, He X, Zhang F, Han J (2018). Exosome-mediated transfer of lncRNA-SNHG14 promotes trastuzumab chemoresistance in breast cancer. Int J Oncol.

[29] Cao YL, Zhuang T, Xing BH, Li N, Li Q (2017). Exosomal DNMT1 mediates cisplatin resistance in ovarian cancer. Cell Biochem Funct.

[30] Shiozawa K, Shuting J, Yoshioka Y, Ochiya T, Kondo T (2018). Extracellular vesicle-encapsulated microRNA-761 enhances pazopanib resistance in synovial sarcoma. Biochem Biophys Res Commun.

[31] Pavlyukov MS, Yu H, Bastola S, Minata M, Shender VO, Lee Y, Zhang S, Wang J, Komarova S, Wang J (2018). Apoptotic cell-derived extracellular vesicles promote malignancy of glioblastoma via intercellular transfer of splicing factors. Cancer Cell.

[32] Zweemer AJM, French CB, Mesfin J, Gordonov S, Meyer AS, Lauffenburger DA (2017). Apoptotic bodies elicit Gas6-mediated migration of AXL-expressing tumor cells. Mol Cancer Res.

[33] Dong L, Pu Y, Zhang L, Qi Q, Xu L, Li W, Wei C, Wang X, Zhou S, Zhu J (2018). Human umbilical cord mesenchymal stem cell-derived extracellular vesicles promote lung adenocarcinoma growth by transferring miR-410. Cell Death Dis.

[34] Ren J, Ding L, Zhang D, Shi G, Xu Q, Shen S, Wang Y, Wang T, Hou Y (2018). Carcinoma-associated fibroblasts promote the stemness and chemoresistance of colorectal cancer by transferring exosomal lncRNA H19. Theranostics..

[35] Zhu L, Kalimuthu S, Gangadaran P, Oh JM, Lee HW, Baek SH, Jeong SY, Lee SW, Lee J, Ahn BC (2017). Exosomes derived from natural killer cells exert therapeutic effect in melanoma. Theranostics..

[36] DeBerardinis RJ, Chandel NS (2016). Fundamentals of cancer metabolism. Sci Adv.

[37] Lehuede C, Dupuy F, Rabinovitch R, Jones RG, Siegel PM (2016). Metabolic plasticity as a determinant of tumor growth and metastasis. Cancer Res.

[38] Pavlides S, Whitaker-Menezes D, Castello-Cros R, Flomenberg N, Witkiewicz AK, Frank PG, Casimiro MC, Wang C, Fortina P, Addya S (2009). The reverse Warburg effect: aerobic glycolysis in cancer associated fibroblasts and the tumor stroma. Cell Cycle.

[39] Kim DK, Lee J, Kim SR, Choi DS, Yoon YJ, Kim JH, Go G, Nhung D, Hong K, Jang SC (2015). EVpedia: a community web portal for extracellular vesicles research. Bioinformatics..

[40] Fong MY, Zhou W, Liu L, Alontaga AY, Chandra M, Ashby J, Chow A, O'Connor ST, Li S, Chin AR (2015). Breast-cancer-secreted miR-122 reprograms glucose metabolism in premetastatic niche to promote metastasis. Nat Cell Biol.

[41] Lazar I, Clement E, Attane C, Muller C, Nieto L (2018). A new role for extracellular vesicles: how small vesicles can feed tumors’ big appetite. J Lipid Res.

[42] Lazaro-Ibanez E, Lunavat TR, Jang SC, Escobedo-Lucea C, Oliver-De La Cruz J, Siljander P, Lotvall J, Yliperttula M (2017). Distinct prostate cancer-related mRNA cargo in extracellular vesicle subsets from prostate cell lines. BMC Cancer.

[43] Zhao H, Yang L, Baddour J, Achreja A, Bernard V, Moss T, Marini J, Tudawe T, Seviour EG, San Lucas FA (2016). Tumor microenvironment derived exosomes pleiotropically modulate cancer cell metabolism. Elife..

[44] Cho J, Lim SI, Yang BS, Hahn YS, Kwon I (2017). Generation of therapeutic protein variants with the human serum albumin binding capacity via site-specific fatty acid conjugation. Sci Rep.

[45] Record M, Carayon K, Poirot M, Silvente-Poirot S (1841). Exosomes as new vesicular lipid transporters involved in cell-cell communication and various pathophysiologies. Biochim Biophys Acta.

[46] Subra C, Grand D, Laulagnier K, Stella A, Lambeau G, Paillasse M, De Medina P, Monsarrat B, Perret B, Silvente-Poirot S (2010). Exosomes account for vesicle-mediated transcellular transport of activatable phospholipases and prostaglandins. J Lipid Res.

[47] Hassani K, Olivier M (2013). Immunomodulatory impact of leishmania-induced macrophage exosomes: a comparative proteomic and functional analysis. PLoS Negl Trop Dis.

[48] Record M, Poirot M, Silvente-Poirot S (2014). Emerging concepts on the role of exosomes in lipid metabolic diseases. Biochimie..

[49] Buschow SI, van Balkom BWM, Aalberts M, Heck AJR, Wauben M, Stoorvogel W (2010). MHC class II-associated proteins in B-cell exosomes and potential functional implications for exosome biogenesis. Immunol Cell Biol.

[50] He M, Qin H, Poon TCW, Sze SC, Ding XF, Co NN, Ngai SM, Chan TF, Wong N (2015). Hepatocellular carcinoma-derived exosomes promote motility of immortalized hepatocyte through transfer of oncogenic proteins and RNAs. Carcinogenesis..

[51] He M, Qin H, Poon TC, Sze SC, Ding X, Co NN, Ngai SM, Chan TF, Wong N. Hepatocellular carcinoma-derived exosomes promote motility of immortalized hepatocyte through transfer of oncogenic proteins and RNAs. Cancer Res. 2015;36:1008–18. 10.1093/carcin/bgv081 26054723

[52] Altamimi TR, Thomas PD, Darwesh AM, Fillmore N, Mahmoud MU, Zhang LY, Gupta A, Al Batran R, Seubert JM, Lopaschuk GD (2018). Cytosolic carnitine acetyltransferase as a source of cytosolic acetyl-CoA: a possible mechanism for regulation of cardiac energy metabolism. Biochem J.

[53] Puhka M, Takatalo M, Nordberg ME, Valkonen S, Nandania J, Aatonen M, Yliperttula M, Laitinen S, Velagapudi V, Mirtti T (2017). Metabolomic profiling of extracellular vesicles and alternative normalization methods reveal enriched metabolites and strategies to study prostate Cancer-related changes. Theranostics..

[54] Hurwitz SN, Rider MA, Bundy JL, Liu X, Singh RK, Meckes DG (2016). Proteomic profiling of NCI-60 extracellular vesicles uncovers common protein cargo and cancer type-specific biomarkers. Oncotarget..

[55] Hong BS, Cho JH, Kim H, Choi EJ, Rho S, Kim J, Kim JH, Choi DS, Kim YK, Hwang D, Gho YS (2009). Colorectal cancer cell-derived microvesicles are enriched in cell cycle-related mRNAs that promote proliferation of endothelial cells. BMC Genomics.

[56] Skog J, Wurdinger T, van Rijn S, Meijer DH, Gainche L, Sena-Esteves M, Curry WT, Carter BS, Krichevsky AM, Breakefield XO (2008). Glioblastoma microvesicles transport RNA and proteins that promote tumour growth and provide diagnostic biomarkers. Nat Cell Biol.

[57] Zhang J, Lu SH, Zhou Y, Meng K, Chen ZP, Cui YZ, Shi YF, Wang T, He QY. Motile hepatocellular carcinoma cells preferentially secret sugar metabolism regulatory proteins via exosomes. Proteomics. 2017;17:13–14. 10.1002/pmic.201700103 28590090

[58] Ronquist KG, Ek B, Stavreus-Evers A, Larsson A, Ronquist G (2013). Human prostasomes express glycolytic enzymes with capacity for ATP production. Am J Physiol Endocrinol Metab.

[59] Ronquist KG, Sanchez C, Dubois L, Chioureas D, Fonseca P, Larsson A, Ullen A, Yachnin J, Ronquist G, Panaretakis T (2016). Energy-requiring uptake of prostasomes and PC3 cell-derived exosomes into non-malignant and malignant cells. J Extracell Vesicles.

[60] Lazar I, Clement E, Dauvillier S, Milhas D, Ducoux-Petit M, LeGonidec S, Moro C, Soldan V, Dalle S, Balor S (2016). Adipocyte exosomes promote melanoma aggressiveness through fatty acid oxidation: a novel mechanism linking obesity and Cancer. Cancer Res.

[61] Ronquist KG (2019). Extracellular vesicles and energy metabolism. Clin Chim Acta.

[62] Qian Y, Wang X, Li Y, Cao Y, Chen X (2016). Extracellular ATP a new player in Cancer metabolism: NSCLC cells internalize ATP in vitro and in vivo using multiple endocytic mechanisms. Mol Cancer Res.

[63] Fischer K, Hoffmann P, Voelkl S, Meidenbauer N, Ammer J, Edinger M, Gottfried E, Schwarz S, Rothe G, Hoves S (2007). Inhibitory effect of tumor cell-derived lactic acid on human T cells. Blood..

[64] Husain Z, Huang Y, Seth P, Sukhatme VP (2013). Tumor-derived lactate modifies antitumor immune response: effect on myeloid-derived suppressor cells and NK cells. J Immunol.

[65] Husain Z, Seth P, Sukhatme VP (2013). Tumor-derived lactate and myeloid-derived suppressor cells: linking metabolism to cancer immunology. Oncoimmunology..

[66] Clayton A, Al-Taei S, Webber J, Mason MD, Tabi Z (2011). Cancer exosomes express CD39 and CD73, which suppress T cells through adenosine production. J Immunol.

[67] Sung BH, Ketova T, Hoshino D, Zijlstra A, Weaver AM (2015). Directional cell movement through tissues is controlled by exosome secretion. Nat Commun.

[68] Hoshino A, Costa-Silva B, Shen TL, Rodrigues G, Hashimoto A, Tesic Mark M, Molina H, Kohsaka S, Di Giannatale A, Ceder S (2015). Tumour exosome integrins determine organotropic metastasis. Nature..

[69] Peinado H, Lavotshkin S, Lyden D (2011). The secreted factors responsible for pre-metastatic niche formation: old sayings and new thoughts. Semin Cancer Biol.

[70] Peinado H, Aleckovic M, Lavotshkin S, Matei I, Costa-Silva B, Moreno-Bueno G, Hergueta-Redondo M, Williams C, Garcia-Santos G, Ghajar CM (2012). Melanoma exosomes educate bone marrow progenitor cells toward a pro-metastatic phenotype through MET. Nat Med.

[71] Costa-Silva B, Aiello NM, Ocean AJ, Singh S, Zhang H, Thakur BK, Becker A, Hoshino A, Mark MT, Molina H (2015). Pancreatic cancer exosomes initiate pre-metastatic niche formation in the liver. Nat Cell Biol.

[72] Umakoshi M, Takahashi S, Itoh G, Kuriyama S, Sasaki Y, Yanagihara K, Yashiro M, Maeda D, Goto A, Tanaka M. Macrophage-mediated transfer of cancer-derived components to stromal cells contributes to establishment of a pro-tumor microenvironment. Oncogene. 2018. 10.1038/s41388-018-0564-x. 10.1038/s41388-018-0564-x 30459356

[73] Zhou W, Fong MY, Min Y, Somlo G, Liu L, Palomares MR, Yu Y, Chow A, O'Connor ST, Chin AR (2014). Cancer-secreted miR-105 destroys vascular endothelial barriers to promote metastasis. Cancer Cell.

[74] Zhang L, Zhang S, Yao J, Lowery FJ, Zhang Q, Huang WC, Li P, Li M, Wang X, Zhang C (2015). Microenvironment-induced PTEN loss by exosomal microRNA primes brain metastasis outgrowth. Nature..

[75] Keklikoglou I, Cianciaruso C, Guc E, Squadrito ML, Spring LM, Tazzyman S, Lambein L, Poissonnier A, Ferraro GB, Baer C, et al. Chemotherapy elicits pro-metastatic extracellular vesicles in breast cancer models. Nat Cell Biol. 2018;21:190–202. 10.1038/s41556-018-0256-3 PMC6525097 30598531

[76] Probert C, Dottorini T, Speakman A, Hunt S, Nafee T, Fazeli A, Wood S, Brown JE, James V. Communication of prostate cancer cells with bone cells via extracellular vesicle RNA; a potential mechanism of metastasis. Oncogene. 2018. 10.1038/s41388-018-0540-5. 10.1038/s41388-018-0540-5 PMC6372071 30353168

[77] Pfeiler S, Thakur M, Grunauer P, Megens RTA, Joshi U, Coletti R, Samara V, Muller-Stoy G, Ishikawa-Ankerhold H, Stark K, et al. CD36-triggered cell invasion and persistent tissue colonization by tumor microvesicles during metastasis. FASEB J. 2019;33:1860–72. 10.1096/fj.201800985R 30207797

[78] Shao Y, Chen T, Zheng X, Yang S, Xu K, Chen X, Xu F, Wang L, Shen Y, Wang T (2018). Colorectal Cancer-derived Small Extracellular Vesicles Establish an Inflammatory Pre-metastatic Niche in Liver Metastasis. Carcinogenesis.

[79] Lafontan M (2012). Historical perspectives in fat cell biology: the fat cell as a model for the investigation of hormonal and metabolic pathways. Am J Phys Cell Phys.

[80] Ouchi N, Parker JL, Lugus JJ, Walsh K (2011). Adipokines in inflammation and metabolic disease. Nat Rev Immunol.

[81] Dirat B, Bochet L, Dabek M, Daviaud D, Dauvillier S, Majed B, Wang YY, Meulle A, Salles B, Le Gonidec S (2011). Cancer-associated adipocytes exhibit an activated phenotype and contribute to breast cancer invasion. Cancer Res.

[82] Crewe C, Joffin N, Rutkowski JM, Kim M, Zhang F, Towler DA, Gordillo R, Scherer PE (2018). An endothelial-to-adipocyte extracellular vesicle Axis governed by metabolic state. Cell..

[83] Vaupel P (2004). Tumor microenvironmental physiology and its implications for radiation oncology. Semin Radiat Oncol.

[84] Kucharzewska P, Christianson HC, Welch JE, Svensson KJ, Fredlund E, Ringner M, Morgelin M, Bourseau-Guilmain E, Bengzon J, Belting M (2013). Exosomes reflect the hypoxic status of glioma cells and mediate hypoxia-dependent activation of vascular cells during tumor development. Proc Natl Acad Sci U S A.

[85] Yang H, Zhang H, Ge S, Ning T, Bai M, Li J, Li S, Sun W, Deng T, Zhang L (2018). Exosome-derived miR-130a activates angiogenesis in gastric Cancer by targeting C-MYB in vascular endothelial cells. Mol Ther.

[86] Chiba M, Kubota S, Sato K, Monzen S (2018). Exosomes released from pancreatic cancer cells enhance angiogenic activities via dynamin-dependent endocytosis in endothelial cells in vitro. Sci Rep.

[87] Ludwig N, Yerneni SS, Razzo BM, Whiteside TL (2018). Exosomes from HNSCC Promote Angiogenesis through Reprogramming of Endothelial Cells. Mol Cancer Res.

[88] Wang S, Xu M, Li X, Su X, Xiao X, Keating A, Zhao RC (2018). Exosomes released by hepatocarcinoma cells endow adipocytes with tumor-promoting properties. J Hematol Oncol.

[89] Yukawa H, Suzuki K, Aoki K, Arimoto T, Yasui T, Kaji N, Ishikawa T, Ochiya T, Baba Y (2018). Imaging of angiogenesis of human umbilical vein endothelial cells by uptake of exosomes secreted from hepatocellular carcinoma cells. Sci Rep.

[90] Tang MKS, Yue PYK, Ip PP, Huang RL, Lai HC, Cheung ANY, Tse KY, Ngan HYS, Wong AST (2018). Soluble E-cadherin promotes tumor angiogenesis and localizes to exosome surface. Nat Commun.

[91] Noy R, Pollard JW (2014). Tumor-associated macrophages: from mechanisms to therapy. Immunity..

[92] Fabbri M, Paone A, Calore F, Galli R, Gaudio E, Santhanam R, Lovat F, Fadda P, Mao C, Nuovo GJ (2012). MicroRNAs bind to toll-like receptors to induce prometastatic inflammatory response. Proc Natl Acad Sci U S A.

[93] Chen X, Zhou J, Li X, Wang X, Lin Y, Wang X (2018). Exosomes derived from hypoxic epithelial ovarian cancer cells deliver microRNAs to macrophages and elicit a tumor-promoted phenotype. Cancer Lett.

[94] Chen X, Ying X, Wang X, Wu X, Zhu Q, Wang X (2017). Exosomes derived from hypoxic epithelial ovarian cancer deliver microRNA-940 to induce macrophage M2 polarization. Oncol Rep.

[95] Buzas EI, Gyorgy B, Nagy G, Falus A, Gay S (2014). Emerging role of extracellular vesicles in inflammatory diseases. Nat Rev Rheumatol.

[96] Lehmann SM, Kruger C, Park B, Derkow K, Rosenberger K, Baumgart J, Trimbuch T, Eom G, Hinz M, Kaul D (2012). An unconventional role for miRNA: let-7 activates toll-like receptor 7 and causes neurodegeneration. Nat Neurosci.

[97] Chow A, Zhou W, Liu L, Fong MY, Champer J, Van Haute D, Chin AR, Ren X, Gugiu BG, Meng Z (2014). Macrophage immunomodulation by breast cancer-derived exosomes requires toll-like receptor 2-mediated activation of NF-kappaB. Sci Rep.

[98] Haderk F, Schulz R, Iskar M, Cid LL, Worst T, Willmund KV, Schulz A, Warnken U, Seiler J, Benner A, et al. Tumor-derived exosomes modulate PD-L1 expression in monocytes. Sci Immunol. 2017;2:eaah5509. 10.1126/sciimmunol.aah5509 28754746

[99] Sumida T, Lincoln MR, Ukeje CM, Rodriguez DM, Akazawa H, Noda T, Naito AT, Komuro I, Dominguez-Villar M, Hafler DA (2018). Activated beta-catenin in Foxp3(+) regulatory T cells links inflammatory environments to autoimmunity. Nat Immunol.

[100] Cerezo M, Guemiri R, Druillennec S, Girault I, Malka-Mahieu H, Shen S, Allard D, Martineau S, Welsch C, Agoussi S (2018). Translational control of tumor immune escape via the eIF4F-STAT1-PD-L1 axis in melanoma. Nat Med.

[101] Wieckowski EU, Visus C, Szajnik M, Szczepanski MJ, Storkus WJ, Whiteside TL (2009). Tumor-derived microvesicles promote regulatory T cell expansion and induce apoptosis in tumor-reactive activated CD8+ T lymphocytes. J Immunol.

[102] Chen G, Huang AC, Zhang W, Zhang G, Wu M, Xu W, Yu ZL, Yang JG, Wang BK, Sun HH (2018). Exosomal PD-L1 contributes to immunosuppression and is associated with anti-PD-1 response. Nature.

[103] Fan Y, Herr F, Vernochet A, Mennesson B, Oberlin E, Durrbach A. Human fetal liver mesenchymal stem cell derived exosomes impair NK cell function. Stem Cells Dev. 2019;28:44–55. 10.1089/scd.2018.0015 30328799

[104] Hanahan D, Weinberg RA (2011). Hallmarks of cancer: the next generation. Cell..

[105] Xu R, Rai A, Chen M, Suwakulsiri W, Greening DW, Simpson RJ (2018). Extracellular vesicles in cancer - implications for future improvements in cancer care. Nat Rev Clin Oncol.

[106] Brenner AW, Su GH, Momen-Heravi F (1882). Isolation of extracellular vesicles for Cancer diagnosis and functional studies. Methods Mol Biol.

[107] Wu AY, Ueda K, Lai CP. Proteomic analysis of extracellular vesicles for cancer diagnostics. Proteomics. 2018;19:e1800162. 10.1002/pmic.201800162 30334355

[108] Han L, Xu J, Xu Q, Zhang B, Lam EW, Sun Y (2017). Extracellular vesicles in the tumor microenvironment: therapeutic resistance, clinical biomarkers, and targeting strategies. Med Res Rev.

[109] Kanada M, Bachmann MH, Contag CH (2016). Signaling by extracellular vesicles advances cancer hallmarks. Trends Cancer.

[110] Mateescu B, Kowal EJ, van Balkom BW, Bartel S, Bhattacharyya SN, Buzas EI, Buck AH, de Candia P, Chow FW, Das S (2017). Obstacles and opportunities in the functional analysis of extracellular vesicle RNA - an ISEV position paper. J Extracell Vesicles.

[111] Chulpanova DS, Kitaeva KV, James V, Rizvanov AA, Solovyeva VV (2018). Therapeutic prospects of extracellular vesicles in Cancer treatment. Front Immunol.

[112] Liu HY, Chen L, Liu JL, Meng HX, Zhang R, Ma L, Wu LL, Yu SY, Shi F, Li Y (2017). Co-delivery of tumor-derived exosomes with alpha-galactosylceramide on dendritic cell-based immunotherapy for glioblastoma. Cancer Lett.

[113] Sun DM, Zhuang XY, Grizzle W, Miller D, Zhang HG. A novel nanoparticle drug delivery system: the anti-inflammatory activity of curcumin is enhanced when encapsulated in exosomes. Mol Ther. 2010;18:1606–14. 10.1038/mt.2010.105 PMC2956928 20571541

[114] Iessi E, Logozzi M, Lugini L, Azzarito T, Federici C, Spugnini EP, Mizzoni D, Di Raimo R, Angelini DF, Battistini L (2017). Acridine Orange/exosomes increase the delivery and the effectiveness of Acridine Orange in human melanoma cells: a new prototype for theranostics of tumors. J Enzyme Inhib Med Chem.

[115] Srivastava A, Amreddy N, Babu A, Panneerselvam J, Mehta M, Muralidharan R, Chen A, Zhao YD, Razaq M, Riedinger N (2016). Nanosomes carrying doxorubicin exhibit potent anticancer activity against human lung cancer cells. Sci Rep.

[116] Kim MS, Haney MJ, Zhao Y, Mahajan V, Deygen I, Klyachko NL, Inskoe E, Piroyan A, Sokolsky M, Okolie O (2016). Development of exosome-encapsulated paclitaxel to overcome MDR in cancer cells. Nanomedicine.

[117] Rivoltini L, Chiodoni C, Squarcina P, Tortoreto M, Villa A, Vergani B, Burdek M, Botti L, Arioli I, Cova A (2016). TNF-related apoptosis-inducing ligand (TRAIL)-armed exosomes deliver Proapoptotic signals to tumor site. Clin Cancer Res.

[118] Lou G, Song X, Yang F, Wu S, Wang J, Chen Z, Liu Y (2015). Exosomes derived from miR-122-modified adipose tissue-derived MSCs increase chemosensitivity of hepatocellular carcinoma. J Hematol Oncol.

[119] Mizrak A, Bolukbasi MF, Ozdener GB, Brenner GJ, Madlener S, Erkan EP, Strobel T, Breakefield XO, Saydam O (2013). Genetically engineered microvesicles carrying suicide mRNA/protein inhibit schwannoma tumor growth. Mol Ther.

[120] Van Deun J, Mestdagh P, Agostinis P, Akay O, Anand S, Anckaert J, Martinez ZA, Baetens T, Beghein E, Bertier L (2017). EV-TRACK: transparent reporting and centralizing knowledge in extracellular vesicle research. Nat Methods.

[121] Van Deun J, Hendrix A, consortium E-T (2017). Is your article EV-TRACKed?. J Extracell Vesicles.

[122] Pathan M, Fonseka P, Chitti SV, Kang T, Sanwlani R, Van Deun J, Hendrix A, Mathivanan S (2019). Vesiclepedia 2019: a compendium of RNA, proteins, lipids and metabolites in extracellular vesicles. Nucleic Acids Res.

[123] Mathivanan S, Simpson RJ (2009). ExoCarta: a compendium of exosomal proteins and RNA. Proteomics..

[124] Kowal J, Arras G, Colombo M, Jouve M, Morath JP, Primdal-Bengtson B, Dingli F, Loew D, Tkach M, Thery C (2016). Proteomic comparison defines novel markers to characterize heterogeneous populations of extracellular vesicle subtypes. Proc Natl Acad Sci U S A.

[125] Mathieu M, Martin-Jaular L, Lavieu G, Thery C (2019). Specificities of secretion and uptake of exosomes and other extracellular vesicles for cell-to-cell communication. Nat Cell Biol.

[126] Yang MQ, Du Q, Goswami J, Varley PR, Chen B, Wang RH, Morelli AE, Stolz DB, Billiar TR, Li J, Geller DA (2018). Interferon regulatory factor 1-Rab27a regulated extracellular vesicles promote liver ischemia/reperfusion injury. Hepatology..

[127] Ji R, Zhang B, Zhang X, Xue J, Yuan X, Yan Y, Wang M, Zhu W, Qian H, Xu W (2015). Exosomes derived from human mesenchymal stem cells confer drug resistance in gastric cancer. Cell Cycle.

[128] Zheng P, Chen L, Yuan X, Luo Q, Liu Y, Xie G, Ma Y, Shen L (2017). Exosomal transfer of tumor-associated macrophage-derived miR-21 confers cisplatin resistance in gastric cancer cells. J Exp Clin Cancer Res.

[129] Min QH, Wang XZ, Zhang J, Chen QG, Li SQ, Liu XQ, Li J, Liu J, Yang WM, Jiang YH (2018). Exosomes derived from imatinib-resistant chronic myeloid leukemia cells mediate a horizontal transfer of drug-resistant trait by delivering miR-365. Exp Cell Res.

[130] Raji GR, Sruthi TV, Edatt L, Haritha K, Shankar SS, Kumar VBS (2017). Horizontal transfer of miR-106a/b from cisplatin resistant hepatocarcinoma cells can alter the sensitivity of cervical cancer cells to cisplatin. Cell Signal.

[131] Wei YF, Lai XF, Yu ST, Chen SN, Ma YZ, Zhang Y, Li HC, Zhu XM, Yao LB, Zhang J (2014). Exosomal miR-221/222 enhances tamoxifen resistance in recipient ER-positive breast cancer cells. Breast Cancer Res Treat.

[132] Wei F, Ma CY, Zhou T, Dong XC, Luo QH, Geng L, Ding LJ, Zhang YD, Zhang L, Li N (2017). Exosomes derived from gemcitabine-resistant cells transfer malignant phenotypic traits via delivery of miRNA-222-3p. Mol Cancer.

[133] Zhang S, Zhang Y, Qu J, Che X, Fan Y, Hou K, Guo T, Deng G, Song N, Li C, et al. Exosomes promote cetuximab resistance via the PTEN/Akt pathway in colon cancer cells. Braz J Med Biol Res. 2018;51:e6472. 10.1590/1414-431X20176472 PMC5685060 29160412

[134] Cocucci E, Meldolesi J (2015). Ectosomes and exosomes: shedding the confusion between extracellular vesicles. Trends Cell Biol.

[135] Choi D, Lee TH, Spinelli C, Chennakrishnaiah S, D'Asti E, Rak J (2017). Extracellular vesicle communication pathways as regulatory targets of oncogenic transformation. Semin Cell Dev Biol.

